# Identification of long noncoding RNAs with aberrant expression in prostate cancer metastases

**DOI:** 10.1530/ERC-22-0247

**Published:** 2023-06-26

**Authors:** Mina Sattari, Annika Kohvakka, Elaheh Moradi, Hanna Rauhala, Henna Urhonen, William B Isaacs, Matti Nykter, Teemu J Murtola, Teuvo L J Tammela, Leena Latonen, G Steven Bova, Juha Kesseli, Tapio Visakorpi

**Affiliations:** 1 Faculty of Medicine and Health Technology, Tampere University and Tays Cancer Center, Tampere University Hospital, Tampere, Finland; 2 A.I. Virtanen Institute for Molecular Sciences, University of Eastern Finland, Kuopio, Finland; 3 The James Buchanan Brady Urological Institute, Johns Hopkins School of Medicine, Baltimore, Maryland, USA; 4 Department of Urology, Tampere University Hospital, Tampere, Finland; 5 Foundation for the Finnish Cancer Institute, Helsinki, Finland; 6 Institute of Biomedicine, University of Eastern Finland, Kuopio, Finland; 7 Fimlab Laboratories Ltd, Tampere University Hospital, Tampere, Finland

**Keywords:** long noncoding RNA, metastatic prostate cancer, arcinoma, etastasis, neoplasia

## Abstract

Prostate cancer (PCa) is the second-most common cause of male cancer-related death in western industrialized countries, and the emergence of metastases is a key challenge in the treatment of PCa. Accumulating studies have shown that long noncoding RNAs (lncRNAs) play an important role in the regulation of diverse cellular and molecular processes during the development and progression of cancer. Here, we utilized a unique cohort of castration-resistant prostate cancer metastases (mCRPC) and corresponding localized tumors and RNA sequencing (RNA-seq). First, we showed that patient-to-patient variability accounted for most of the variance in lncRNA expression between the samples, suggesting that genomic alterations in the samples are the main drivers of lncRNA expression in PCa metastasis. Subsequently, we identified 27 lncRNAs with differential expression (DE-lncRNAs) between metastases and corresponding primary tumors, suggesting that they are mCRPC-specific lncRNAs. Analyses of potential regulation by transcription factors (TFs) revealed that approximately half of the DE-lncRNAs have at least one binding site for the androgen receptor in their regulatory regions. In addition, TF enrichment analysis revealed the enrichment of binding sites for PCa-associated TFs, such as FOXA1 and HOXB13, in the regulatory regions of the DE-lncRNAs. In a cohort of prostatectomy-treated prostate tumors, four of the DE-lncRNAs showed association with progression-free time and two of them (lnc-SCFD2-2 and lnc-R3HCC1L-8) were independent prognostic markers. Our study highlights several mCRPC-specific lncRNAs that might be important in the progression of the disease to the metastatic stage and may also serve as potential biomarkers for aggressive PCa.

## Introduction

Prostate cancer (PCa) is one of the most common cancers in men. Localized PCa is curable by radiation therapy or surgery. For advanced disease, androgen deprivation therapy (ADT) has been the golden standard treatment for more than half decade. However, ADT is non-curative, and once the disease progresses it is called castration-resistant prostate cancer (CRPC). During the last 20 years, several new treatment options for CRPC have been developed. These include chemotherapy, immunotherapy, radiotherapy, and especially novel androgen receptor (AR) signaling suppressors. However, all these treatment modalities are non-curative and allow only the progression of the disease to be slowed and symptoms to be alleviated (Cornford *et al.* 2021). The most common metastatic sites in PCa are bone, lungs, pleura, lymph nodes, liver, and adrenal glands (Bubendorf *et al.* 2000).

Long noncoding RNAs (lncRNAs) are a group of RNA molecules that have a length greater than 200 nucleotides and lack protein-coding potential. lncRNAs may regulate a wide range of biological and pathological processes, including the development of cancer (Wilusz *et al.* 2009; Schmitt & Chang 2016). lncRNAs function in tumorigenesis and cancer progression through transcriptional or post-transcriptional regulation of protein-coding genes (Geisler & Coller 2013). RNA-seq analyses of numerous normal and cancer tissues have revealed differential expression of various lncRNAs in tumor tissues, indicating that aberrant expression of lncRNAs might be implicated in tumor progression (Yan *et al.* 2015). In PCa, aberrant lncRNA expression has been reported to be involved in PCa development, metastasis, and prognosis (Hua *et al.* 2018, Mitobe *et al.* 2018, Wu *et al.* 2020, Zhang *et al.* 2020). For example, multiple lncRNAs, such as PCA3, a well-known example of PCa-specific lncRNAs, have been found to be related to PCa (Bussemakers *et al.* 1999, De Kok *et al.* 2002, Martens-Uzunova *et al.* 2014, Kohvakka *et al.* 2020). As most lncRNAs have tissue- and cancer-specific expression patterns, they are excellent candidates as biomarkers for cancer diagnosis and prognosis as well as potential therapeutic targets for cancer treatment (Sahu *et al.* 2015, Misawa *et al.* 2017).

We have previously identified a number of PCa-associated lncRNAs by comparing benign prostatic hyperplasia, untreated PCa, and locally recurrent CRPCs (Ylipää *et al.* 2015). Here, we aimed to identify lncRNAs that are associated with metastatic CRPC by utilizing a unique sample set of primary tumors and CRPC metastases (mCRPCs) from the same patients. We identified several PCa metastasis-specific lncRNAs and indicated multiple transcriptional regulators for them. Using various RNA-seq datasets from several PCa cell lines, we addressed the androgen regulation of the differentially expressed (DE)-lncRNAs, the effect of AR coregulator silencing, and the association of DE-lncRNAs to enzalutamide resistance. Additionally, we evaluated the prognostic value of the metastasis-specific lncRNAs in a dataset of 81 untreated PCa tumors. The identified DE-lnRNAs may shed light on the biology of PCa metastasis and serve as novel biomarkers.

## Materials and methods

### mCRPC samples, prostatectomy, and cell line samples and sequencing

mCRPC cohort consisted of 106 samples obtained from autopsies of 25 PCa patients with metastatic castration-resistant disease (Supplementary Table 1, see section on supplementary materials given at the end of this article). Of the 25 patients, six had primary tumor samples available: five patients had one primary tumor sample and one patient had two. The metastases were from different sites: liver (*n* = 12), adrenal gland (*n* = 7), bone (*n* = 18), subdural (*n* = 6), and lymph node (*n* = 40). Normal samples from the liver (*n* = 14) and adrenal gland (*n* = 2) from 15 patients in our cohort were also included. In addition, normal samples from other tissue types in the mCRPC cohort, with the exception of the lymph node, were retrieved from publicly available datasets. Data are available in European Genome-phenome Archive (EGA) under accession number EGAS00001006959.

Prostatectomy cohort consisted of freshly frozen tissue specimens from 81 untreated PCas from Tampere University Hospital (Tampere, Finland). Data can be accessed using accession number EGAD00001009996 in the EGA database.

PCa cell line LNCaP was obtained from American Type Cell Collection (ATCC), and VCaP cell line was kindly provided by Dr Jack Schalken (Radboud University Nijmegen Medical Center, Nijmegen, the Netherlands). Detailed description of the samples, library construction, sequencing, alignment, and expression quantification can be found in supplementary materials and methods. Data can be accessed using accession number GSE223024 at the NCBI GEO database.

Detailed descriptions of the samples, RNA sequencing, alignment, and expression quantification can be found in the Supplementary material and methods with sample visualizations (Supplementary Fig. 1 and 2).

### Data visualization

For visualization and clustering of RNA-seq data of the mCRPC cohort, we used variance stabilizing transformation counts calculated by DESeq2 (version 1.22.2) (Love *et al.* 2014), and PCA plots were generated. The PCA plots were generated for a smaller part of the dataset, including 32 samples from the six patients who had samples from primary tumors in addition to metastases.

### Pipeline for finding metastasis-associated lncRNAs

To identify DE-lncRNAs between primary prostate tumors and metastases, gene expression profiles were compared using a four-stage design.

#### Differential expression analysis

Differential expression analysis between primary and metastatic tumors was performed using DESeq2 R package (Love *et al.* 2014). The Benjamini–Hochberg method was used for multiple testing correction to obtain adjusted *P*-values. lncRNAs with an adjusted *P*-value of less than 0.15 were considered differentially expressed. A relaxed cutoff was used for the first step of selection to obtain a larger set of lncRNAs for the subsequent screening steps.

#### Filtering with tissue-specific controls

To remove the lncRNAs whose differential expression was due to their location in a specific tissue, we performed three different filtering steps with tissue-specific controls. First, all primary tumors were compared separately to each normal tissue outside the prostate to remove those lncRNAs whose observed differential expression between primary tumors and metastases could be attributed to the higher basal expression in a given non-prostate tissue. Second, all normal samples outside the prostate were compared against all of the primary tumors to remove those lncRNAs whose observed differential expression between primary tumors and metastases could be attributed to the overall higher expression in tissues other than the prostate. Third, the non-malignant benign prostatic hyperplasia (BPH) samples were used as normal prostate tissue controls. A comparison between BPH samples and all other normal tissues together was performed to remove those lncRNAs whose observed differential expression between primary tumors and metastases could be attributed to the differential expression between BPH and normal tissues. In addition, in each step, the direction of the expression change was checked for the lncRNAs. Those lncRNAs whose expression changes were not in opposite directions in controls relative to primary tumors or BPH samples were kept. Differential expression analysis using DESeq2 was used for the filtering steps. For the tissue-specific filtering steps, an adjusted *P*-value of less than 0.01 was chosen as the threshold for statistical significance.

#### Pairwise analysis

The directions of expression change of the remaining non-tissue-specific DE-lncRNAs were checked in six patients with both primary and metastatic tumors by calculating the mean expression values of DE-lncRNAs in metastatic tumors for each patient and comparing them against the expression value of their corresponding primary tumor from the same patient. For the patient with two primary tumors, the means of expression values of DE-lncRNAs in both primary and metastatic tumors were calculated separately and compared against each other. lncRNAs that displayed expression changes in opposite directions in more than two patients were removed from the list of DE-lncRNAs of interest.

#### Overlaps with protein-coding genes or pseudogenes and manual curation

DE-lncRNAs with same-strand and at least one base pair overlap with any coordinates annotated as protein-coding genes or pseudogenes were considered to be overlapping and were selected for manual curation. Annotations of coding genes and pseudogenes were derived from Ensemble 99 (Yates *et al.* 2020) and the GENCODE (https://www.gencodegenes.org) basic annotation files, respectively. For manual curation, Integrative Genomics Viewer (IGV 2.5.0) was used to visualize the pattern of DE-lncRNA alignments in the LNCipedia v.5.2 high-confidence annotation set. DE-lncRNAs that did not overlap with either protein-coding genes or pseudogenes were kept, as were those DE-lncRNAs that overlapped in part with protein-coding genes or pseudogenes, but their alignment pattern was clearly in concordance with the LNCipedia v5.2 high-confidence annotation set.

### Analysis of transcription factor (TF) regulation of DE-lncRNAs

Homo sapiens meta-cluster ChIP-sequencing (chromatin immunoprecipitation sequencing) data were downloaded from the Gene Transcription Regulation Database (GTRD) (Yevshin *et al.* 2019). For enrichment analysis, we investigated whether the binding sites of given TFs were enriched within the regulatory regions (-5 kb/+2 kb from the transcription start site (TSS)) of the DE-lncRNAs. One-tailed Fisher's exact test was used to test for the enrichment of the binding sites for a given TF in the regulatory regions of the DE-lncRNAs relative to the regulatory regions of all lncRNAs in the dataset. An adjusted *P*-value of less than 0.15 was used as a statistical cutoff for enriched TFs. The read counts in our mCRPC cohort were normalized using the transcripts per kilobase million method in order to visualize the expression levels of the enriched TFs in primary tumors and metastases.

For transcription factor-binding site analysis, AR, FOXA1, and HOXB13 ChIP-seq peaks in human prostate tumor tissues were retrieved from a publicly available dataset (GSE56288) (Pomerantz *et al.* 2015). We counted the number of AR, FOXA1, and HOXB13 peaks in the regulatory regions of DE-lncRNAs. A minimum of one base pair overlap was required between a peak and the regulatory region.

### Regulation of lncRNAs by AR and its coregulators and their association with enzalutamide resistance

To study the androgen effects on DE-lncRNAs, we analyzed our RNA-seq data from LNCaP and VCaP cells with AR siRNA knockdown and dihydrotestosterone (DHT) stimulation. To identify FOXA1- and HOXB13-regulated lncRNAs, RNA-seq data from VCaP cells transfected with non-targeting (NSI) or FOXA1 targeting siRNA (siFOXA1) (GSE193127) and from LNCaP cells infected with control shRNA (pGIPZ) or shRNA targeting HOXB13 (shHOXB13) (GSE153585) were retrieved from publicly available datasets. To investigate the expression of DE-lncRNAs in enzalutamide-resistant and sensitive cells, we used our previously published RNA-seq data from androgen-sensitive VCaP (VCAP-T) and enzalutamide-resistant VCaP (VCaP-CT-ER) (GSE169305). Differential expression analysis using DESeq2 was performed between treatment and control groups of each cell line. An adjusted *P*-value of less than 0.05 was used as a threshold for statistical significance. Prostate-specific antigen PSA (KLK3), an androgen-responsive gene, was used as a positive control.

### Survival analysis and multivariant Cox regression analysis

Kaplan–Meier survival analysis was performed in a prostatectomy cohort (*n* = 81) to evaluate the prognostic significance of DE-lncRNAs. The third quartile was used as a threshold for high vs low expression. A Cox-proportional hazards model was used to evaluate the association between progression-free survival and multiple factors, such as age at diagnosis, PSA levels, and pathologic T status (pT) (Supplementary Table 2). Gleason score (GS) variable violated the proportional hazards assumption of the Cox regression model; therefore, we used a model stratified for GS, but it was not a variable in the model itself.

Age was divided into two categories: low (age under 62) and high (age above 62). Similarly, pT was divided into low (pT stages between 2 and 4) and high (pT stages 5 and 6). Diagnostic PSA values were divided into three categories: low (PSA ≤10), intermediate (PSA from 10 to 19.9), and high (PSA >20). GSs were also divided into three groups: low (GS < 7), intermediate (GS = 7), and high (GS > 7).

## Results

### PCa metastases show large variability in lncRNA expression

**Figure 1:**
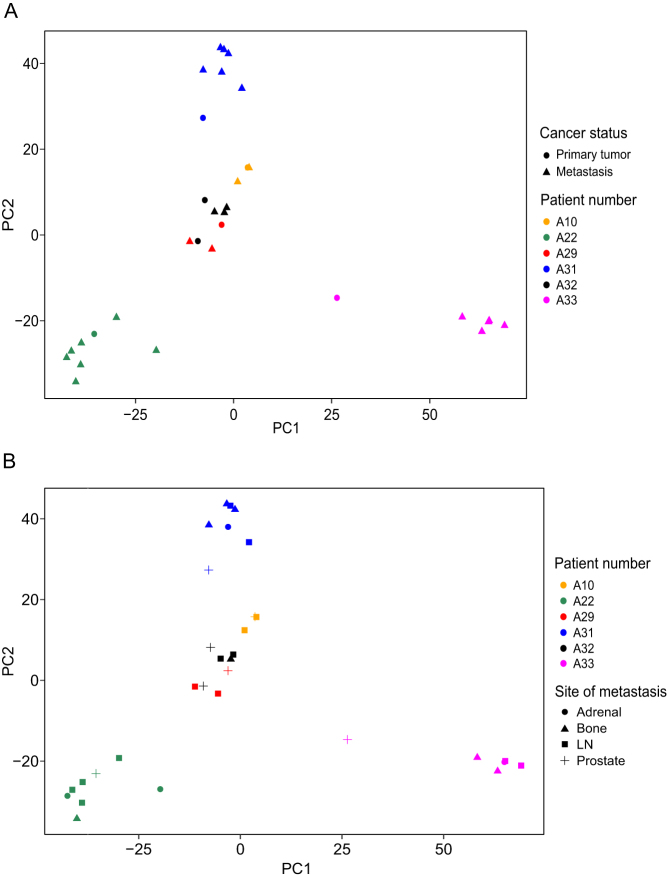
Principal component analysis (PCA) of primary and metastatic tumors. Patients with samples from primary tumors and metastases were selected to generate the PCA plots. (A) PCA of RNA-seq data for patient numbers and cancer status covariates; patient numbers are color coded, and cancer status (primary tumor – metastasis) is shape coded. (B) PCA of RNA-seq data for patient numbers and sites of metastases covariates; patient numbers are color coded and sites of metastases are shape coded. A full color version of this figure is available at https://doi.org/10.1530/ERC-22-0247.

To discover and characterize lncRNAs clinically relevant to the progression of PCa to metastatic disease, we compared the expression of 42,371 lncRNAs from LNCipedia v. 5.2 high-confidence annotation set (hg38) in PCa samples of primary tumors and metastases. First, principal component analysis (PCA) was used to visualize the overall influence of empirical covariates of cancer status (normal, primary, or metastatic tumor), patient number, and site of metastasis on lncRNA expression. Two PCA plots were generated using a subset of the lncRNA expression data, namely, the data from patients who had samples from both primary tumors and metastases (Fig. 1). The PCA plots showed that samples were mainly clustered based on the patient number, and there was no noticeable clustering of samples according to the primary vs metastasis status or the site of the metastasis. These results suggest that patient-to-patient variability accounts for most of the variance in lncRNA expression in primary tumors and metastases of PCa.

### Identification of PCa metastasis-specific lncRNAs

**Figure 2:**
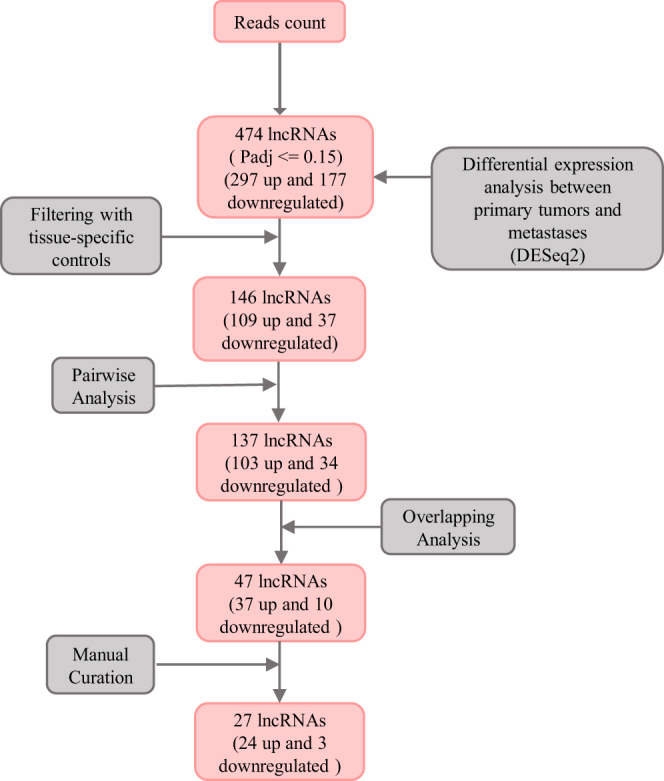
Pipeline for RNA-seq analysis to discover metastatic prostate cancer-associated lncRNAs. Differential expression analysis followed by filtering with tissue-specific controls, pairwise analysis, overlapping analysis, and manual curation. A full color version of this figure is available at https://doi.org/10.1530/ERC-22-0247.

Differential expression analysis was performed between primary tumors and metastases. In total, 474 lncRNAs were identified as DE-lncRNAs between primary tumors and metastases with an adjusted *P* < 0.15. Of these, 297 lncRNAs were significantly upregulated, and 177 were downregulated in metastases compared with primary tumors (Fig. 2). Previous studies have shown that lncRNAs have a higher tissue specificity than protein-coding genes (Cabili *et al.* 2011, Brunner *et al.* 2012). Therefore, to test whether the differential expression of the identified DE-lncRNAs in metastases was associated with the metastatic sites (tissue types), we investigated the tissue specificity of our DE-lncRNAs in three steps, as described in the ‘Materials and methods’ section. After filtering out tissue-specific DE-lncRNAs, 146 lncRNAs showed putative associations with PCa metastasis (Fig. 2).

As patient-to-patient variability was large in our lncRNA dataset (Fig. 1), a pairwise analysis was performed for the remaining non-tissue-specific DE-lncRNAs to reduce the effect of the patient variability. Based on the analysis, of 146 DE-lncRNAs, 9 showed non-consistent expression changes between patients and were excluded from the previous findings, leaving 137 DE-lncRNAs (Fig. 2).

Next, overlap analysis and IGV visualization were performed, and 110 DE-lncRNAs were omitted based on expression possibly attributed to protein coding or pseudogenes, leaving 27 DE-lncRNAs. Of these, 24 lncRNAs were upregulated and 3 were downregulated in metastases (Fig. 2).

**Figure 3:**
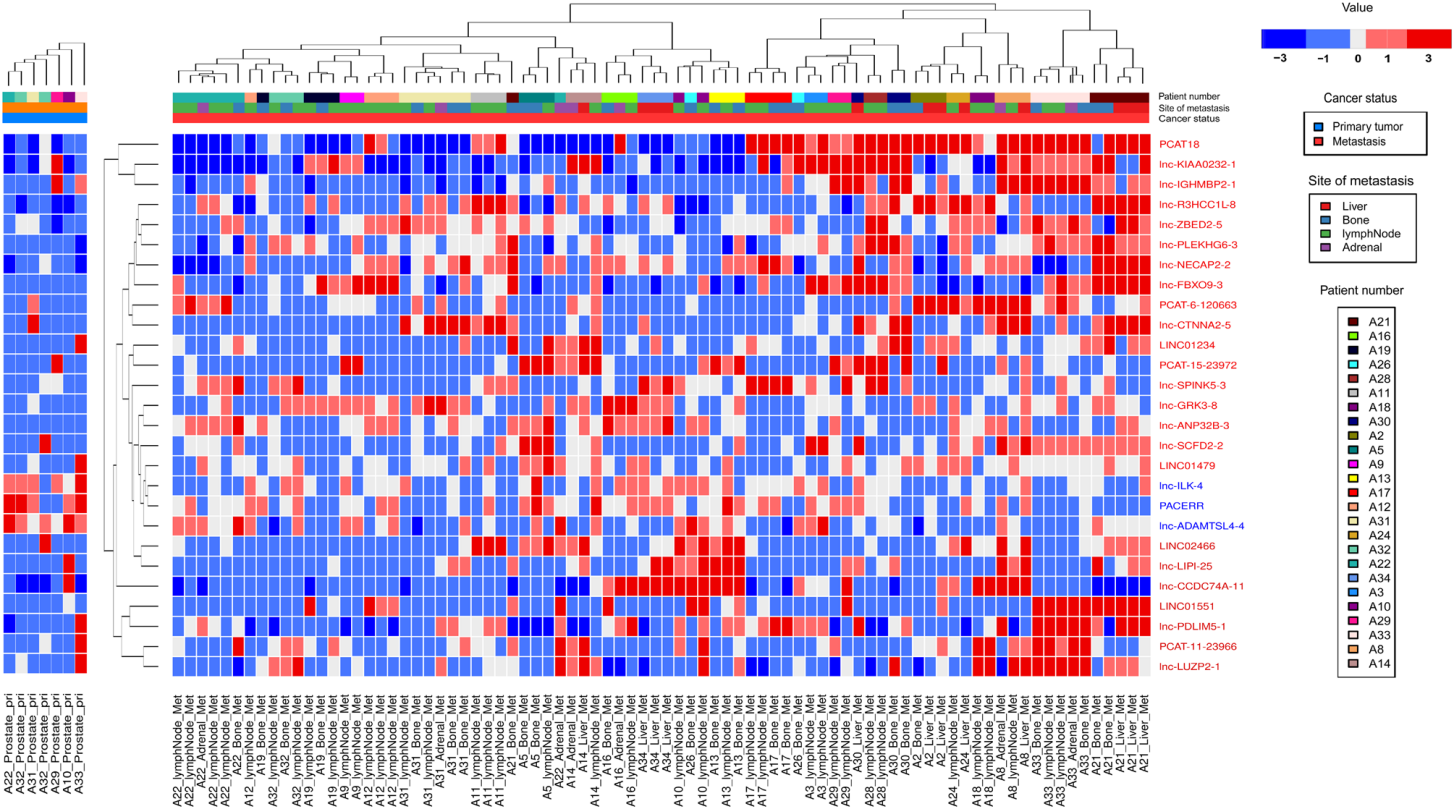
Heatmap of DE-lncRNAs between primary and metastatic tumors. Heatmap showing the expression levels of DE-lncRNAs across metastatic PCa samples (right) and primary PCa samples (left). The color bars at the top indicate the different variables in the dataset, patient numbers, site of metastasis, and cancer status. The expression value of each DE-lncRNA is represented in a color scale, where red indicates high relative expression and blue indicates low relative expression. The DE-lncRNAs were clustered based on their expression across metastatic PCa samples. The lncRNA names are colored based on whether they were upregulated (red) or downregulated (blue). A full color version of this figure is available at https://doi.org/10.1530/ERC-22-0247.

A heatmap generated from the expression of these 27 DE-lncRNAs is shown in Fig. 3. Due to the strong effect of patient variability on sample clustering, two heatmaps were generated separately for primary and metastatic tumors. Gene clustering was performed based on the expression of the 27 DE-lncRNAs in metastatic tumors. Even though samples were mainly clustered based on patient IDs in metastatic samples, some tissue-specific clustering was also observed within patients.

### Analysis of TFs regulating lncRNA expression in PCa metastasis

**Figure 4:**
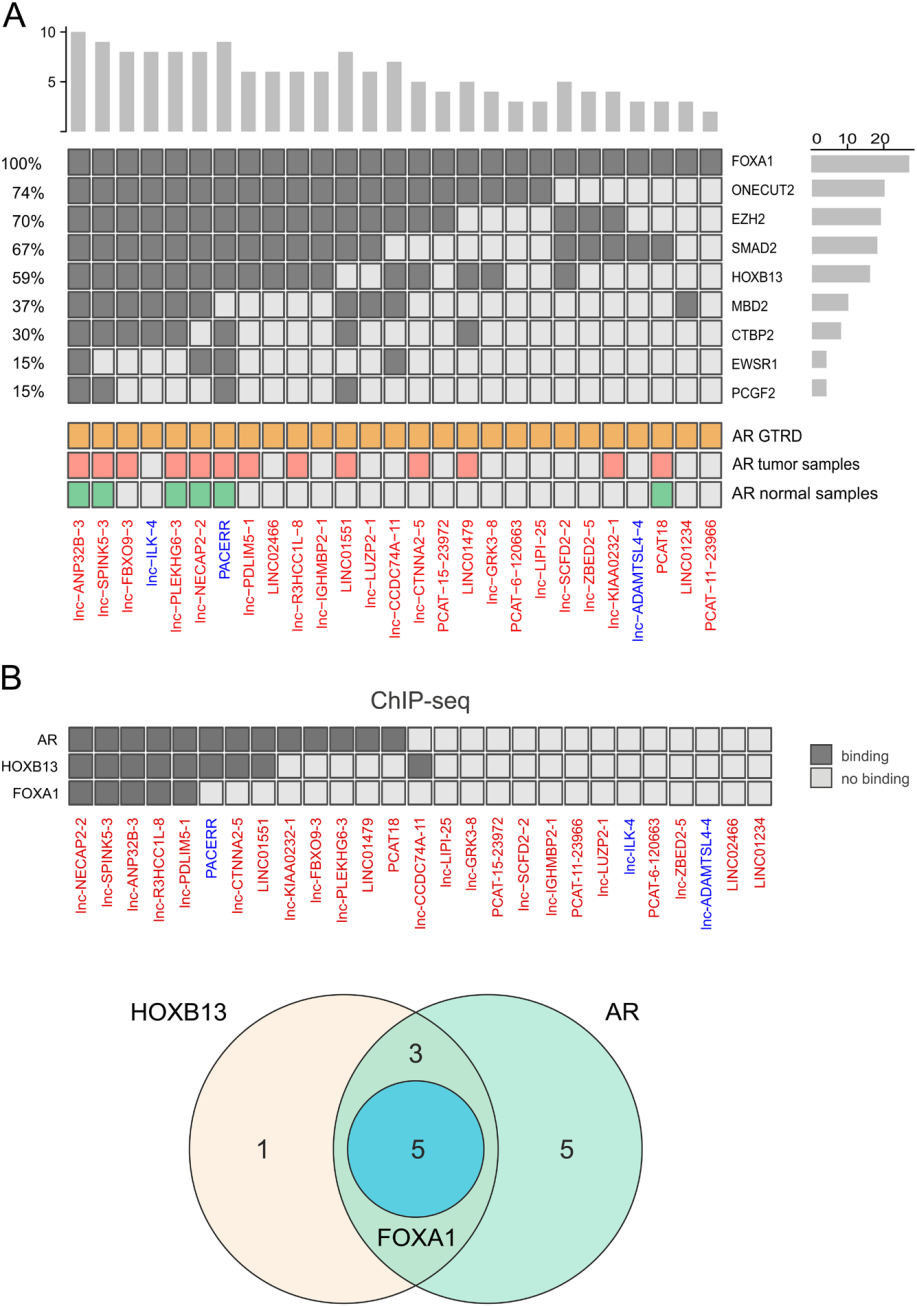
Transcription factors enriched in the regulatory regions of DE-lncRNAs. (A) The heatmap indicates the presence of binding sites for the nine TFs in the regulatory regions (−5 kb/+2 kb from TSS) of DE-lncRNAs using ChIP-seq peaks in the GTRD database. The dark gray color represents binding sites for each TF, and light gray represents no binding site. TFs were ordered in decreasing order of their frequencies. The percentage on the left shows the fraction of DE-lncRNAs with a binding site for the given TF. The *Y*-axis on the right side of the heatmap shows the number of lncRNAs with a binding site for the given TF. The histogram data above the heatmap show the number of TFs binding per lncRNA. The lower part of the heatmap shows the ChIP-seq-derived AR-binding sites (ARBS) in the regulatory regions of the DE-lncRNAs in the GTRD database and previously published prostate tumor and normal prostate tissue-specific ARBS. The lncRNA names are colored based on whether they are upregulated (red) or downregulated (blue). (B) Upper: The heatmap shows the existence of binding sites for AR, FOXA1, and HOXB13 in the regulatory regions of DE-lncRNAs. Lower: The Venn diagram indicates the number of DE-lncRNAs with ChIP-seq peaks detected for AR, FOXA1, and HOB13 in their regulatory regions (−5 kb/+2 kb from TSS). Previously published AR, FOXA1, and HOXB13 ChIP-seq data in PC tumor samples were used for this purpose. A full color version of this figure is available at https://doi.org/10.1530/ERC-22-0247.

To identify putative transcriptional regulators of the 27 DE-lncRNAs, transcription factor enrichment analysis (TFEA) was performed on the regulatory regions (-5 kb/+2kb) of the DE-lncRNAs using the GTRD. In total, binding sites of nine key TFs were significantly enriched within the regulatory regions of the DE-lncRNAs (Fig. 4A). All nine TFs, including FOXA1, HOXB13, EZH2, ONECUT2, SMAD2, MBD2, CTBP2, EWSR1, and PCGF2, have been previously reported to be associated with PCa (Srikantan *et al.* 2000, Varambally *et al.* 2002, Perttu *et al.* 2006, Pulukuri & Rao 2006, Ewing *et al.* 2012, Gerhardt *et al.* 2012, Takayama *et al.* 2014, Guo *et al.* 2019, Nicholas *et al.* 2021). The number of binding sites for each enriched TF is presented in Supplementary Table 3. AR, a TF that plays a central role in PCa, did not show enrichment at the promoter regions of DE-lncRNAs, but the result showed that all 27 DE-lncRNAs had at least one AR-binding site in their promoter region according to the GTRD database.

Since the high expression of AR is important for the progression of PCa to CRPC (Linja *et al.* 2001), we further investigated the AR binding in the regulatory regions of DE-lncRNAs by utilizing previously published prostate tumor and prostate normal tissue-specific AR-binding sites (ARBS) (Pomerantz *et al.* 2015). We found that 48% of the DE-lncRNAs (13 out of 27) had at least one tumor-specific ARBS in their regulatory regions, while only 22% (6 out of 27) showed a normal prostate-specific ARBS (Fig. 4A, Supplementary Table 4). The total number of tumor-specific ARBSs in the regulatory regions of the DE-lncRNAs was approximately four times higher than that of the normal prostate-specific ARBSs (Supplementary Table 4). It has been previously shown that FOXA1 and HOXB13 are pioneer factors directing AR function in PCa (Pomerantz *et al.* 2015). Therefore, to identify whether FOXA1 and HOXB13 are colocalized with AR in the regulatory regions (−5 kb/+2 kb from TSS) of the DE-lncRNAs, we used previously published FOXA1 and HOXB13 ChIP-seq data in PCa tumor samples (Pomerantz *et al.* 2015). Of the 13 prostate tumor-specific ARBSs, five were co-occupied by both FOXA1 and HOXB13 (Fig. 4B), suggesting that FOXA1 and HOXB13 also contribute to the regulation of our DE-lncRNAs in PCa. To provide additional illustration, we employed the same previously published ChiP-seq data to align the AR, FOXA1, and HOXB13 peaks around the regulatory regions of lnc-R3HCC1L-8, one of our DE-lncRNAs. The analysis demonstrated co-occupation of ARBS by FOXA1 and/or HOXB13, which implies the potential for regulatory interactions involving these factors (Supplementary Fig. 3).

To determine the role of these TFs in metastases, the expression levels of the enriched TFs together with AR were investigated in our cohort of primary tumors and metastases. The results revealed that the master regulators in PCa, namely, AR, FOXA1, and HOXB13, were expressed at high levels in the majority of the samples (Supplementary Fig. 4). In addition, the other TFs with binding sites in the regulatory regions of the DE-lncRNAs (Supplementary Fig. 4) were among the 25% of the most highly expressed genes, supporting their potential role as regulators of metastasis-associated lncRNAs in PCa.

To further assess androgen regulation of the DE-lncRNAs, we investigated the expression of the DE-lncRNAs by RNA-seq in LNCaP and VCaP cells treated with 0 nM or 10 nM DHT as well as in AR-silenced cells (Supplementary Table 5). The data indicated a significant downregulation for two of the metastasis-upregulated DE-lncRNAs, lnc-LUZP2-1, and PCAT-11-23966 in LNCaP cells upon DHT stimulation. However, no changes in the expression of these DE-lncRNAs were found in AR-siRNA-treated LNCaP cells. Similar to our observations with LNCaP cells, DHT stimulation downregulated lnc-LUZP2-1 and PCAT-11-23966 in VCaP cells. In addition, two other lncRNAs, lnc-GRK3-8 and lnc-ZBED2-5, showed downregulation and four lncRNAs, PCAT18, lnc-FBXO9-3, lnc-PLEKHG6-3, and lnc-CCDC74A-11, showed upregulation in VCaP cells upon DHT stimulation. AR knockdown resulted in the upregulation of lnc-LUZP2-1 in VCaP cells (Supplementary Table 5).

To evaluate FOXA1 or HOXB13 regulation on the expression of our DE-lncRNAs, we utilized publicly available RNA-seq data (Del Giudice *et al.* 2022, Lu *et al.* 2022). For both TFs, we performed differential expression analysis between controls and knockdown cells (siFOXA1 or shHOXB13). Results showed upregulation of three DE-lncRNAs, lnc-PDLIM5-1, lnc-LIPI-25, and LINC02466, and downregulation of six DE-lncRNAs, PCAT-11-23966, lnc-LUZP2-1, lnc-R3HCC1L-8, lnc-ANP32B-3, lnc-CCDC74A-11, and lnc-NECAP2-2, upon FOXA1 silencing (*P* < 0.05). HOXB13 silencing resulted in the upregulation of PCAT18 and downregulation of two of the DE-lncRNAs (lnc-LUZP2-1 and lnc-ZBED2-5) (*P* < 0.05) (Supplementary Table 6).

### Identification of enzalutamide-resistance associated DE-lncRNAs

We used our previously published RNA-seq data (Taavitsainen *et al.* 2021) to assess the expression of DE-lncRNAs in enzalutamide-sensitive (VCaP-CT) and resistant VCaP cell line (VCaP-CT-ER) (Taavitsainen *et al.* 2021). Four DE-lncRNAs (lnc-CCDC74A-11, lnc-LUZP2-1, PCAT-11-23966, and lnc-LIPI-25) showed upregulation (*P* < 0.05) and four lncRNAs (LINC02466, lnc-R3HCC1L-8, LINC01234, and PCAT18) showed downregulation (*P* < 0.05) in enzalutamide-resistant cells (Supplementary Table 7).

### Association of the DE-lncRNAs with progression-free survival of PCa patients

**Figure 5:**
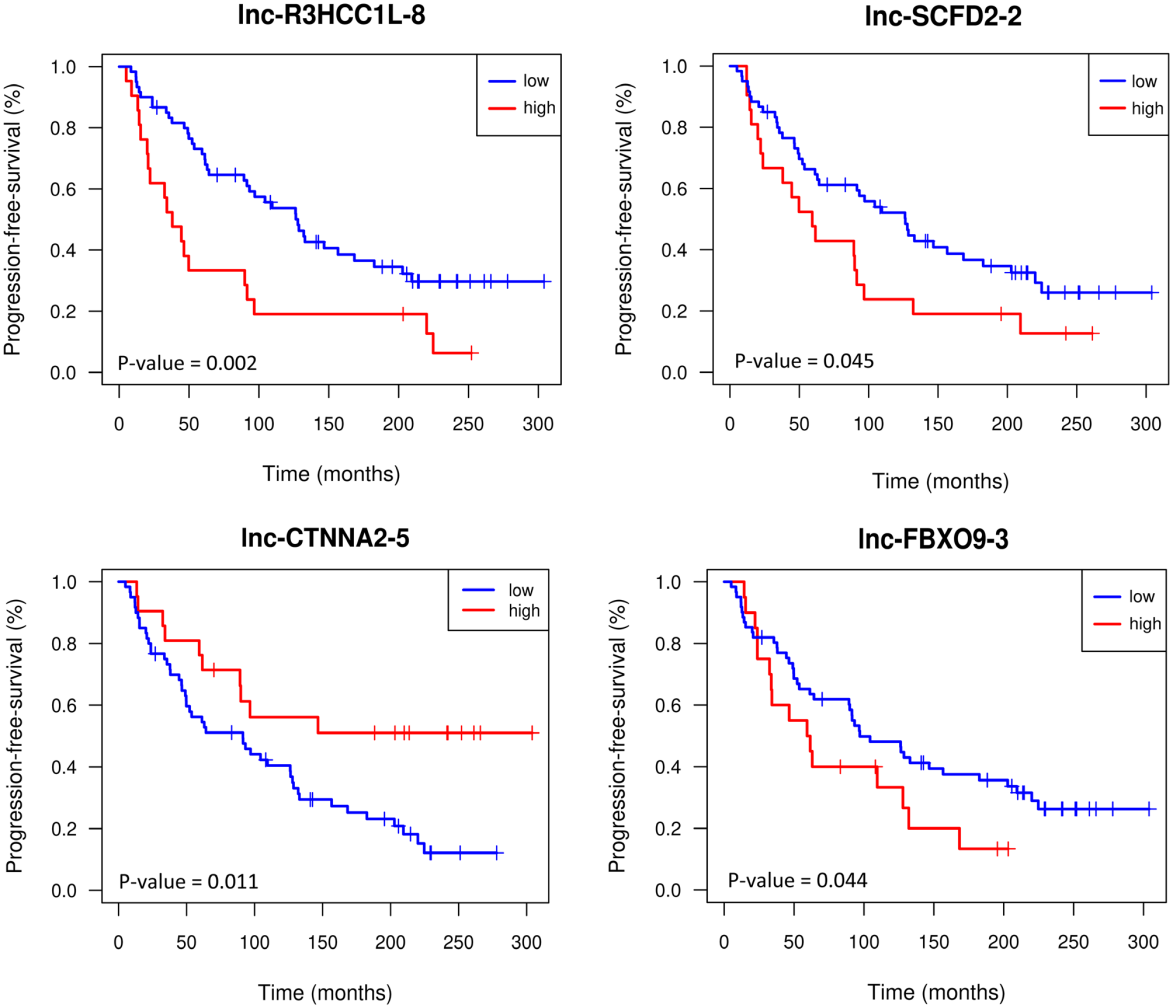
Survival curves of the prostatectomy cohort study. Kaplan–Meier analysis was used to study the progression-free survival of PCa patients. The third quartile was used as a threshold for high vs low expression. *P*-values were calculated by log-rank test. A full color version of this figure is available at https://doi.org/10.1530/ERC-22-0247.

To investigate the potential of the metastasis-associated DE-lncRNAs as prognostic biomarkers, we assessed the association of their expression with the progression-free survival in 81 prostatectomy-treated patients. Kaplan–Meier analysis was performed between patient groups with high- vs low-expression levels of the DE-lncRNAs. With a *P*-value of less than 0.05, we found that expression levels of lnc-SCFD2-2, lnc-FBXO9-3, lnc-R3HCC1L-8, and lnc-CTNNA2-5 were associated with progression-free time (Fig. 5). High-expression levels of lnc-SCFD2-2, lnc-FBXO9-3, and lnc-R3HCC1L-8 were associated with poor prognosis, whereas a high expression of lnc-CTNNA2-5 was associated with better prognosis (Fig. 5). Furthermore, we employed multivariate Cox regression analysis to assess the independence of those DE-lncRNAs as prognostic markers. Results revealed that the expression of lnc-SCFD2-2 and lnc-R3HCC1L-8 were independent predictors for biochemical recurrence (Table 1). The hazard ratios for lnc-SCFD2-2 and lnc-R3HCC1L-8 in the univariate analyses are given in the Supplementary Table 8.

**Table 1:** Multivariate Cox regression analysis. The third quartile was used as cutoff point for high vs low expression. The mean of the patient’s age was 62.

	lnc-SCFD2-2		lnc-R3HCC1L-8	
Variable	HR (95% CI)	*P*-value	HR (95% CI)	*P*-value
Low EXP level	1.000 (reference)		1.000 (reference)	
High EXP level	1.994 (1.063–3.741)	0.031	2.080 (1.008 - 4.291)	0.047
PSA ≤10	1.000 (reference)		1.000 (reference)	
PSA from 10 to 19.9	1.246 (0.652–2.384)	0.504	0.966 (0.470–1.987)	0.926
PSA >20	4.673 (1.939–11.263)	0.0005	3.342 (1.440–7.757)	0.0049
Age ≤ 62	1.000 (reference)		1.000 (reference)	
Age > 62	1.521 (0.818–2.829)	0.184	1.562 (0.843–2.893)	0.155
pT levels from 2 to 4	1.000 (reference)		1.000 (reference)	
pT levels 5 and 6	1.774 (1.015–3.099)	0.043	1.852 (1.061–3.231)	0.029

HR, hazard ratio; pT, pathological T stage.

The associations of the expression of these these DE-lncRNA with age, GS, diagnostic PSA, and pT are shown in Supplementary Fig. 5.

## Discussion

With recent advances in sequencing technologies, a large number of lncRNAs have been discovered to be associated with PCa (Srikantan *et al.* 2000, Chung *et al.* 2011, Prensner *et al.* 2013). For example, we recently identified 145 novel PCa-associated lncRNAs, some of which are specific for localized CRPC (Ylipää *et al.* 2015). Here, we specifically aimed to identify CRPC metastasis-specific lncRNAs. We investigated the aberrant expression of lncRNAs in metastasis to increase our understanding of lncRNA expression changes during the progression of cancer to metastatic disease, which is the main cause of patient mortality.

Our sample set of both multiple metastases and primary tumors provided us with a unique opportunity to identify PCa metastasis-related lncRNAs. The PCA of the expression profile of lncRNAs revealed that samples were mainly clustered by the patient and not by the site of metastasis, which implies that the expression of lncRNAs in metastases is mostly driven by the tumor genome and not by the environment of the metastasis. This is in concordance with our previous findings on the genomics of these same samples, indicating that genomic aberrations are mainly shared between the metastases and the primary tumor in a given patient (Liu *et al.* 2009, Gundem *et al.* 2015). However, despite the strong effect of the patient-related genetic background in our lncRNA dataset, we were able to identify 27 candidate lncRNAs whose regulation was altered due to metastasis. Of these, 24 were transcriptionally upregulated and 3 were downregulated. Of the 27 DE-lncRNAs, three lncRNAs, LINC01551, PACER, and PCAT18, have previously been reported to be important in various cancers, including PCa (Crea *et al.* 2014, Krawczyk & Emerson 2014, Gao *et al.* 2019). PCAT18 (Prostate Cancer-Associated Transcript-18) is a known androgen-regulated lncRNA specifically expressed in PCa that plays an important role in PCa progression and has the potential as a putative target for PCa treatment (Crea *et al.* 2014). The remaining DE-lncRNAs represent functionally uncharacterized lncRNAs.

We also explored the mechanisms driving the expression of the 27 PCa metastasis-related DE-lncRNAs and showed enrichment of nine TFs within their regulatory regions. Interestingly, all of the identified TFs, namely, FOXA1, HOXB13, EZH2, SMAD2, MBD2, ONECUT2, CTBP2, EWSR1, and PCGF2, have been previously reported to be associated with PCa, highlighting common transcriptional regulation between protein-coding genes and long non-coding RNAs in PCa (Srikantan *et al.* 2000, Varambally *et al.* 2002, Perttu *et al.* 2006, Pulukuri & Rao 2006, Ewing *et al.* 2012, Gerhardt *et al.* 2012, Takayama *et al.* 2014, Guo *et al.* 2019, Nicholas *et al.* 2021).

AR is one of the critical TFs for PCa tumorigenesis (Heinlein & Chang 2004). In earlier studies, several lncRNAs, such as ARLNC1 (Zhang *et al.* 2018), PCAT18 (Crea *et al.* 2014), PCAT29 (Malik *et al.* 2014), and EPCART (Kohvakka *et al.* 2020), were identified as AR-regulated lncRNAs, and their association with PCa has been shown. In this study, TFEA did not show enrichment of AR within the regulatory regions of these 27 DE-lncRNAs compared to lncRNAs in general, even though all 27 DE-lncRNAs had binding sites for AR. Thus, ARBS are commonly found in the regulatory regions of all lncRNAs, not only for those that show aberrant expression in PCa metastasis. In agreement with the TFEA results, analyzing specific publicly available AR-ChIP-seq data from human prostate tumors and normal tissues revealed that 48% of our DE-lncRNAs contained at least one tumor-specific AR-binding site within their regulatory regions, while this percentage was smaller for normal prostate-specific ARBS (22%).

Furthermore, in two androgen-sensitive PCa cell lines, LNCaP and VCaP, two of the metastasis-upregulated DE-lncRNAs, lnc-LUZP2-1 and PCAT-11-23966, showed androgen responsiveness. Treatment with DHT caused a significant reduction in their expressions. We next considered whether decreasing the expression of FOXA1 and HOXB13, well-known AR coregulators, affects the expression of the DE-lncRNAs. The knockdown of FOXA1 in VCaP resulted in the upregulation of lnc-PDLIM5-1, lnc-LIPI-25, and LINC02466 and in the downregulation of PCAT-11-23966, lnc-LUZP2-1, lnc-R3HCC1L-8, lnc-ANP32B-3, lnc-CCDC74A-11, and lnc-NECAP2-2. Knockdown of HOXB13 resulted in the upregulation of PCAT18 and downregulation of lnc-LUZP2-1 and lnc-ZBED2-5. The association of DE-lncRNAs to enzalutamide resistance was also examined, and eight DE-lncRNAs showed differential expression in enzalutamide-resistant compared to sensitive cells, namely lnc-CCDC74A-11, lnc-LUZP2-1, PCAT-11-23966, and lnc-LIPI-25 (upregulated) and LINC02466, lnc-R3HCC1L-8, and LINC01234, and PCAT18 (downregulated). While interpreting all of these cell line experiments, one has to take into account that the expression levels of these lnRNAs in the cell lines are low. Thus, these models are not optimal to study their regulation by TFs or by enzalutamide resistance. However, these results imply that AR-mediated mechanisms together with other key TFs, such as AR cofactors FOXA1 and HOXB13, contribute to the aberrant expression of the DE-lncRNAs in PCa metastasis.

Four of the 27 CRPC metastasis-associated DE-lncRNAs, lnc-SCFD2-2, lnc-FBXO9-3, lnc-R3HCC1L-8, and lnc-CTNNA2-5, showed prognostic value in the prostatectomy-treated patient cohort. Two of these, lnc-SCFD2-2 and lnc-R3HCC1L-8, were independent markers of shorter progression-free time. The function of neither these DE-lncRNAs is known, and thus, further studies are required to understand their value as clinically useful biomarkers and their mechanistic significance.

In summary, we identified 27 DE-lncRNAs between primary prostate tumors and CRPC metastases. Based on the TF-binding site analyses, the majority of the DE-lncRNAs seem to be regulated by previously reported PCa-related TFs, such as AR and its coregulators FOXA1 and HOXB13. In a separate primary PCa dataset, four DE-lncRNAs showed significant prognostic value, and two of them were found to be independent prognostic markers. Altogether, our findings indicate novel lncRNAs that may play a role in the progression of PCa and potentially serve as novel biomarkers. Further experiments are needed to evaluate the mechanistic and prognostic significance of these lncRNAs in PCa.

## Supplementary Materials

Supplementary Material

Supplementary Figures

Supplementary Table. S1. Table showing the number of samples from each patient and tissue in our lncRNA cohort.

Supplementary Table S2: Clinical data of prostate cancer patients treated with radical prostatectomy.

Supplementary Table. S3. Table of the number of binding sites and p-values for the 9 enriched TFs can be included.

Supplementary Table. S4. Table of AR binding sites in Pomerantz dataset.

Supplementary Table S5: Non-normalized read counts of 27 DE-lncRNAs. PSA (KLK3) was used as a positive control.

Supplementary Table S6: Result table for differential expression analysis between VCaP cells transfected with non-targeting (NSI) and FOXA1 targeting siRNA (siFOXA1).

Supplementary Table S7: Result table for differential expression analysis between androgen-independent VCaP (VCAP-CT) and enzalutamide-resistant VCaP (VCaP-CT-ER).

Supplementary Table S8. Univariate Cox regression analysis.

## Declaration of interest

The authors declare that there is no conflict of interest that could be perceived as prejudicing the impartiality of the research reported.

## Funding

This work was supported by the Translational Research Network for Prostate Cancer (TransPot) and was funded by the European Union’s Horizon 2020 research and innovation program under the Marie Skłodowska-Curie grant agreement No 721746 (TV). Additionally, this research was supported by the Academy of Finland (TV 279270; LL 317871; MN 312043, GSB 294820), Sigrid Juselius Foundation (TV, LL, GSB, MN, TT), the Cancer Foundation Finland (TV, LL, GSB, MN, TM), the Competitive State Research Financing of the Expert Responsibility area of Tampere University Hospital (TV, TT, TJM) and the Nordic Cancer Union (TJM).
