# Supplementary material for: Identification of long noncoding RNAs with aberrant expression in prostate cancer metastases
Source: Endocr Relat Cancer. 2023 Jun 26;30(8):e220247. doi: 10.1530/ERC-22-0247 (PMC10326635; doi:10.1530/ERC-22-0247)
Supplement: Supplementary Material [file supplementary_material.pdf]

mCRPC sample cohort

Metastatic CRPC specimens were obtained from men who participated in the PELICAN (Project to ELIminate lethal CANcer) integrated clinical-molecular autopsy study of metastatic prostate cancer. Subjects consented to participate in the Johns Hopkins Medicine IRB-approved study between 1995 and 2005. Exhaustive curated phenotypic information including drug exposure data for the PELICAN mCRPC cohort is contained in Jasu et al PMID 34337548 (Jasu *et al.* 2021) and is deposited in the European Genome-Phenome Archive under EGAS00001005399.

In total, samples from 6 tissue types (prostate, lymph node, adrenal gland, liver, subdural, and bone) were used. Notably, in our RNA-seq data, 6 samples were subdural. As subdural is not a specific tissue type but an area between the brain tissue and the bony skull, principal component analysis (PCA) and hierarchical clustering were used to determine whether subdural could be categorized as bone tissue (Supplementary Fig. 1). A heatmap of sample-to-sample distances was generated to visualize the Similarities and differences between bone and subdural metastases (Supplementary Fig. 1). The PCA plot and the heatmap did not show distinct clustering for bone or subdural metastases; therefore, subdural metastases were classified as bone metastases.

A PCA plot and heatmap of the sample-to-sample distances were used to detect outliers among the remaining samples in the dataset. Based on Supplementary Fig. 2A and B, the cancer sample A21-LClavicleLNMet-20194 was considered an outlier and removed from the dataset.

RNA-seq sample preparation, library construction, and sequencing for mCRPC cohort

Tissue blocks embedded in OCT (Optimal Cutting Temperature compound, Tissue-Tek) were serial −20 °C cryostat microdissected for histological tumor purity >75%. Noncancerous samples were without histologically detectable tumors. During serial dissection, OCT compound was trimmed away completely before the isolation of sections for RNA extraction. HMW DNA was isolated from 50 to 500 six micron sections per dissected tissue block using Qiagen All-Prep Universal Kit Catalog Number 80224 using manufacturer's instructions. Library preparation and RNA-seq from frozen cryostat-microdissected tissue was performed using 250-300 bp insert cDNA libraries and PE150 mRNA seq (Illumina, Novogene). Data are available in European Genome-phenome Archive (EGA) under accession number EGAS00001006959.

Publicly available data acquisition and analysis

For tissue specificity analysis, normal samples from each tissue type in the RNA-seq data, with the exception of the lymph node, were used as controls. Our dataset contained 14 samples of normal liver. RNA-seq data for normal bone were retrieved from the publicly available dataset GSE72815 (Farr et al. 2015) (14 samples). In addition to 2 normal adrenal gland samples in our cohort, RNA-seq data for 4 normal adrenal glands from GSE88668 (Dunham et al. 2012) and GSE88157 (Dunham et al. 2012) were retrieved. Benign prostatic hyperplasia (BPH) samples (EGAS00001000526) (Annala et al. 2015) were used as controls for the prostate tissue (7 samples). All publicly available RNA-seq data files were downloaded in FASTQ format.

RNA-seq alignment and expression quantification

FastQC tool v. 0.11.7 was used for the quality control analysis of RNA-seq samples. Alignment of RNA-seq reads was performed using STAR v. 2.5.2b, v. 2.5.3a, and v. 2.7.1a (Dobin *et al.* 2013) for different datasets used in this study. Alignment files were indexed using SAMtools (Li *et al.* 2009). To quantify lncRNA expression levels in the various datasets, a custom lncRNA annotation file was created by combining LNCipedia (Volders *et al.* 2019) high confidence annotation set (hg38) version 5.2 with PCA novel transcripts (Ylipää *et al.* 2015) that were lifted over from hg19 to hg38 using the UCSC liftOver web tool (Hinrichs *et al.* 2006). LncRNA transcripts that belonged to the same gene were merged to obtain gene-level counts. The strandedness of the samples was checked with RSeQC v. 4.0.0 (Wang *et al.* 2012).

Quantification of protein-coding and lncRNA transcripts for mCRPC cohort was performed with Kallisto v. 0.46.0 (Bray *et al.* 2016). For that, a Kallisto transcriptome index was built from the Ensembl GRCh38 cDNA FASTA file (Howe *et al.* 2021), LNCipedia v. 5.2 high-confidence annotation set (hg38) FASTA file (Volders *et al.* 2019) and a FASTA file of PCa novel transcripts that was converted from a BED file with the UCSC twoBitToFa command-line tool. BEDTools v. 2.27.1 sub-command multicov (Quinlan & Hall 2010) was used for read quantification in prostatectomy and cell line samples.

Prostatectomy sample cohort

Prostatectomy cohort consisted of freshly frozen tissue specimens from 81 untreated prostate cancers from Tampere University Hospital (Tampere, Finland). Untreated PCa

samples were obtained by radical prostatectomy, snap frozen, and stored in liquid nitrogen. Histological evaluation and Gleason grading were performed by a pathologist based on hematoxylin/eosin-stained slides. All samples contained a minimum of 70% cancerous cells. The mean age at diagnosis was 62 years (range: 47.7–71.8) and the mean PSA at diagnosis was 11.3 ng/ml (range: 1.7–51.5) (Supplementary Table S2). The study has been approved by the Ethical Board of Tampere University Hospital, and the patients had given written informed consent.

Cell lines and treatments

Prostate cancer cell line LNCaP was obtained from American Type Cell Collection (ATCC, Manassas, VA, USA), and VCaP cell line was kindly provided by Dr. Jack Schalken (Radboud University Nijmegen Medical Center, Nijmegen, the Netherlands). The cells were cultured as recommended by the suppliers and tested for mycoplasma contamination regularly.

To identify AR-regulated lncRNAs, LNCaP and VCaP cells were treated with 0 or 10 nM of dihydrotestosterone (DHT) for 24 hours and knocked down with AR siRNA or negative control siRNA for 48 hours as previously described (Kohvakka *et al.* 2020).

RNA-seq sample preparation, library construction, and sequencing for cell lines and prostatectomy samples

RNA from both cell lines (three biological replicates) and prostatectomy samples was isolated using Trizol (Invitrogen, Thermo Fisher Scientific), treated with RNase-free DNase set (Qiagen), and purified by Monarch RNA Cleanup Kit (New England Biolabs) according to manufacturers’ protocols. The purified RNA was quantified by Qubit 4 Fluorometer

(Invitrogen, Thermo Fisher Scientific) and Qubit RNA Broad Range Assay Kit (Invitrogen, Thermo Fisher Scientific), and its purity was assessed by the 260nm/280nm ratio. RNA integrity was checked using the 5300 Fragment Analyzer System (Agilent Technologies). Library preparation was performed using standard polyA enrichment and sequenced with a Novaseq 6000 (Illumina) in Novogene (Hong Kong, China) for 150 bp paired-end reads. The library was prepared as strand-specific for prostatectomy samples, but not for cell line samples. On average, 105 million raw reads per sample were obtained from prostatectomy samples and 24 million raw reads per sample from cell line samples.

Supplementary references

Bray NL, Pimentel H, Melsted P & Pachter L 2016 Near-optimal probabilistic RNA-seq quantification. *Nature Biotechnology 2016 34:5* **34** 525–527. (doi:10.1038/nbt.3519)

Dobin A, Davis CA, Schlesinger F, Drenkow J, Zaleski C, Jha S, Batut P, Chaisson M & Gingeras TR 2013 STAR: Ultrafast universal RNA-seq aligner. *Bioinformatics* **29** 15–21. (doi:10.1093/bioinformatics/bts635)

Hinrichs AS, Karolchik D, Baertsch R, Barber GP, Bejerano G, Clawson H, Diekhans M, Furey TS, Harte RA, Hsu F *et al.* 2006 The UCSC Genome Browser Database: update 2006. *Nucleic Acids Research* **34**. (doi:10.1093/nar/gkj144)

Howe KL, Achuthan P, Allen J, Allen J, Alvarez-Jarreta J, Ridwan Amode M, Armean IM, Azov AG, Bennett R, Bhai J *et al.* 2021 Ensembl 2021. *Nucleic Acids Research* **49** D884. (doi:10.1093/NAR/GKAA942)

Jasu J, Tolonen T, Antonarakis ES, Beltran H, Halabi S, Eisenberger MA, Carducci MA, Loriot Y, Van der Eecken K, Lolkema M *et al.* 2021 Combined Longitudinal Clinical and Autopsy Phenomic Assessment in Lethal Metastatic Prostate Cancer: Recommendations for

Advancing Precision Medicine. *European Urology Open Science* **30** 47. (doi:10.1016/J.EUROS.2021.05.011)

Kohvakka A, Sattari M, Shcherban A, Annala M, Urbanucci A, Kesseli J, Tammela TLJ, Kivinummi K, Latonen L, Nykter M *et al.* 2020 AR and ERG drive the expression of prostate cancer specific long noncoding RNAs. *Oncogene* **39** 5241–5251. (doi:10.1038/S41388-020-1365-6)

Li H, Handsaker B, Wysoker A, Fennell T, Ruan J, Homer N, Marth G, Abecasis G & Durbin R 2009 The Sequence Alignment/Map format and SAMtools. *Bioinformatics* **25** 2078–2079. (doi:10.1093/bioinformatics/btp352)

Volders PJ, Anckaert J, Verheggen K, Nuytens J, Martens L, Mestdagh P & Vandesompele J 2019 Lncipedia 5: Towards a reference set of human long non-coding rnas. *Nucleic Acids Research* **47** D135–D139. (doi:10.1093/nar/gky1031)

Wang L, Wang S & Li W 2012 RSeQC: Quality control of RNA-seq experiments. *Bioinformatics* **28** 2184–2185. (doi:10.1093/bioinformatics/bts356)

Ylipää A, Kivinummi K, Kohvakka A, Annala M, Latonen L, Scaravilli M, Kartasalo K, Leppänen SP, Karakurt S, Seppäla J *et al.* 2015 Transcriptome sequencing reveals PCAT5 as a Novel ERG-Regulated long Noncoding RNA in prostate cancer. *Cancer Research* **75** 4026–4031. (doi:10.1158/0008-5472.CAN-15-0217)
