# Supplementary material for: Identification of long noncoding RNAs with aberrant expression in prostate cancer metastases
Source: Endocr Relat Cancer. 2023 Jun 26;30(8):e220247. doi: 10.1530/ERC-22-0247 (PMC10326635; doi:10.1530/ERC-22-0247)
Supplement: Supplementary Figures [file supplementary_figure_1.pdf]

## Supplementary Figures

A

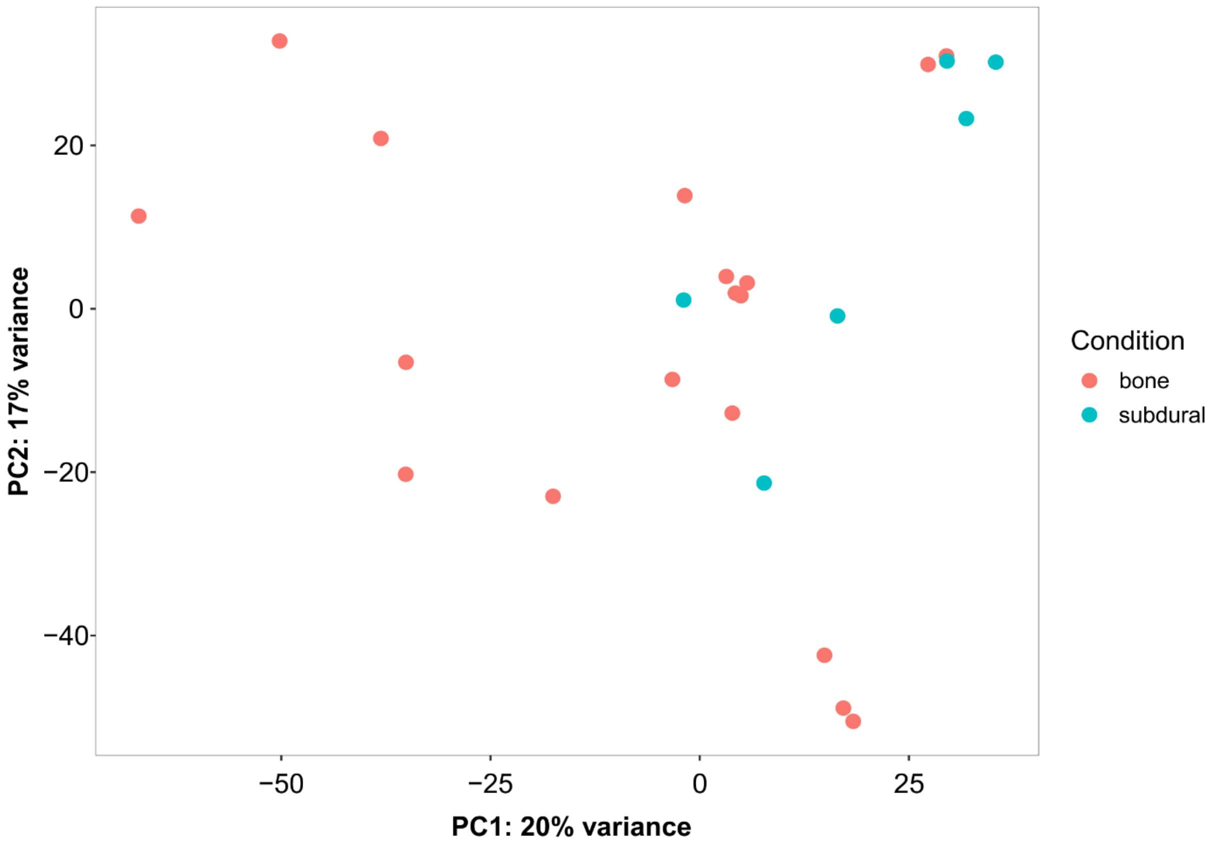


B

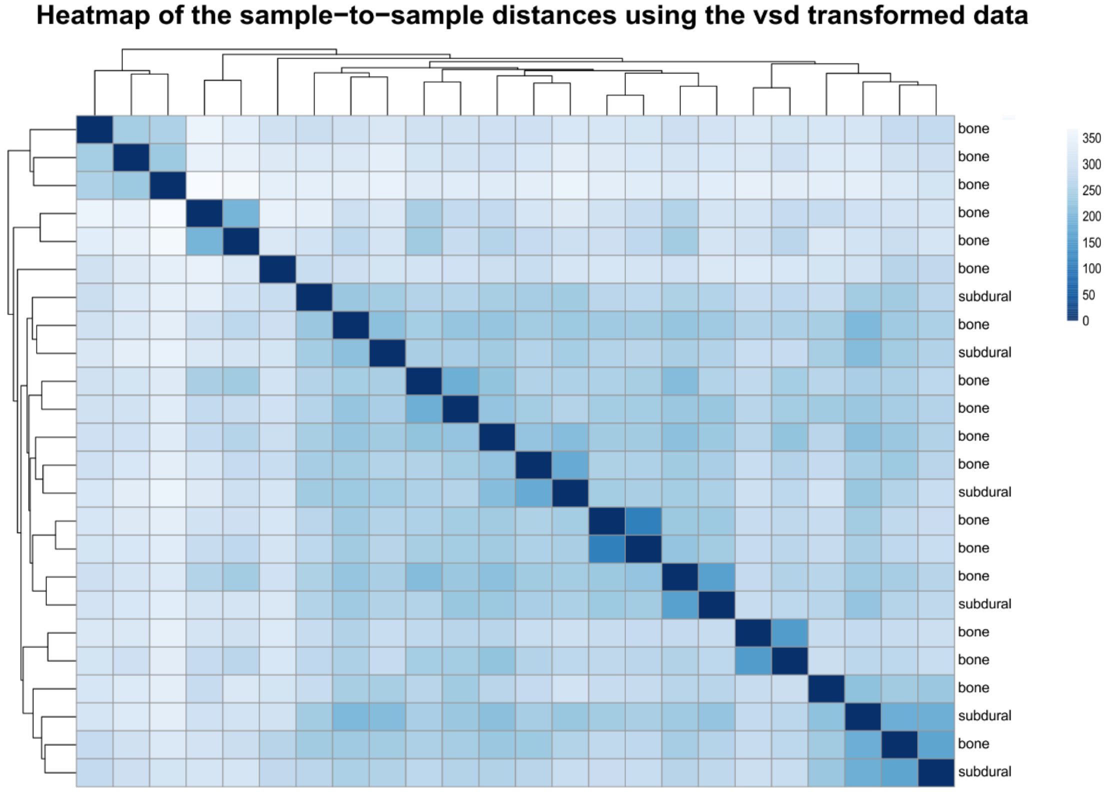

**Supplementary Fig. 1. Principal component analysis (PCA) and hierarchical clustering of bone and subdural metastases in our sample cohort.**

A PCA plot and a heatmap of sample-to-sample distances were generated using DESeq2 on the matrix of variance-stabilized data (VSD). **(A)** PCA plot; samples are color-coded, where red indicates bone metastases, and blue indicates subdural metastases. A clear separation between bone and subdural metastases could not be detected. **(B)** Heatmap of sample-to-sample distances; darker blue colors demonstrate more similar expression and lighter blue colors demonstrate less similar expression between bone and subdural metastases.

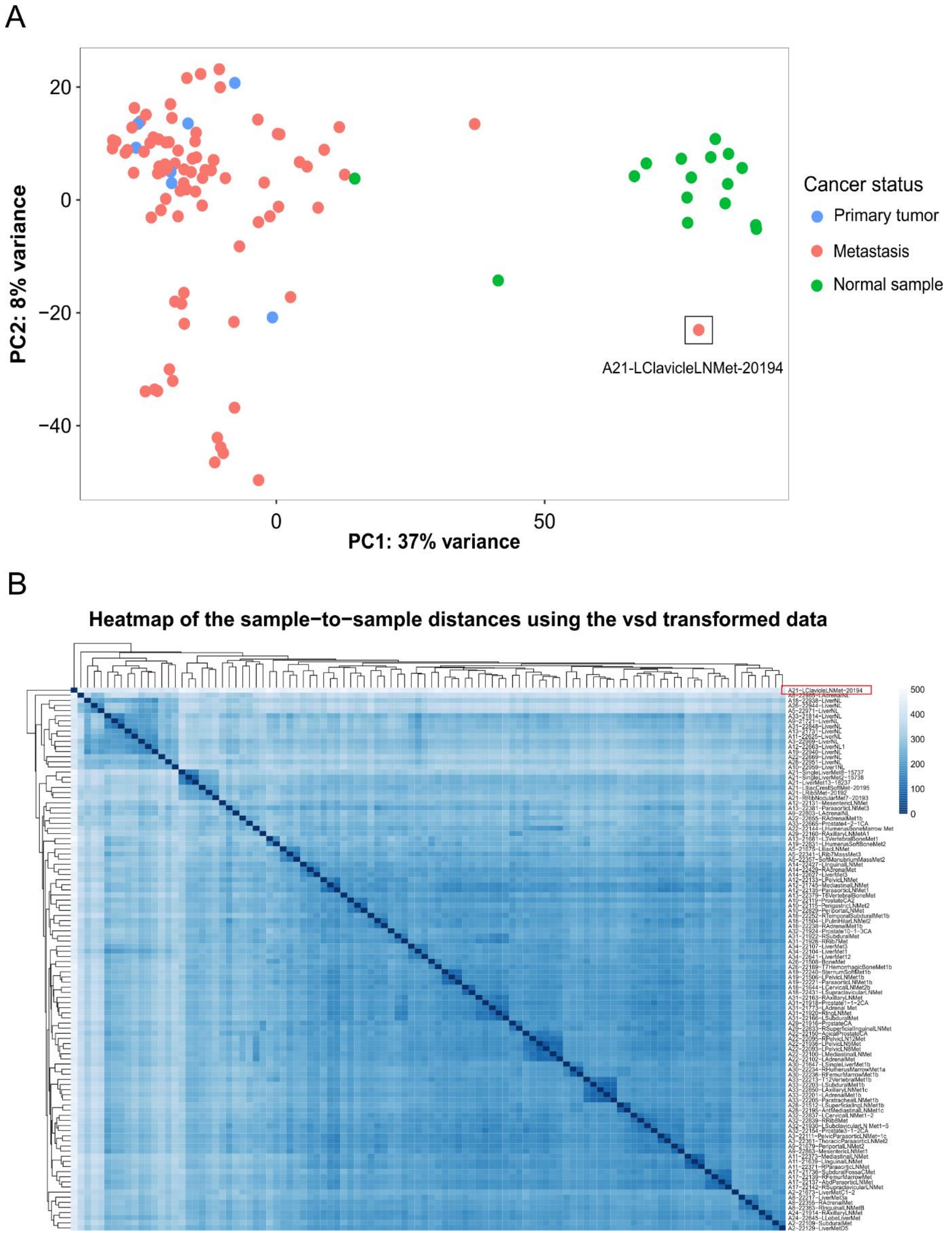
A
20
0
−20
−40
0
50
PC1: 37% variance
PC2: 8% variance
Cancer status
Primary tumor
Metastasis
Normal sample
A21-LClavicleLNMet-20194
B
Heatmap of the sample−to−sample distances using the vsd transformed data
500
400
300
200
100
0

**Supplementary Fig. 2. Principal component analysis (PCA) and hierarchical clustering of our lncRNA cohort.**

**(A)** PCA of the lncRNA expression data for primary tumors, metastases, and normal samples; cancer status is color-coded. **(B)** The heatmap of sample-to-sample distances was built using DESeq2 on the matrix of variance-stabilized data (VSD). Darker blue colors indicate more similar expression and lighter blue colors indicate less similar expression between the samples. The cancer sample,  A21-LClavicleLNMet-20194, showed complete separation from the other cancers and was considered an outlier and excluded from further analyses.

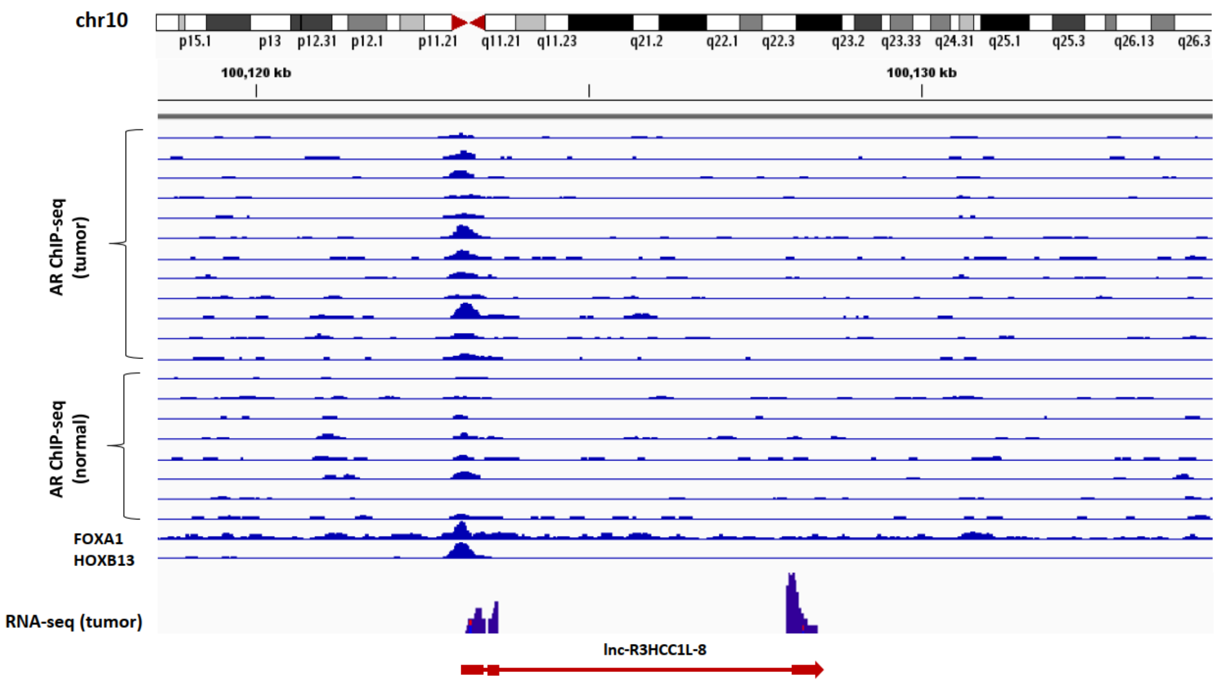

**Supplementary Fig. 3. Potential regulatory interactions of lncRNA lnc-R3HCC1L-8 with AR, FOXA1, and HOXB13 in prostate cancer.**

The binding sites for AR, FOXA1, and HOXB13 in the regulatory region of lnc-R3HCC1L-8 were determined using publicly available ChIP-seq data. Furthermore, the transcript structure of the lncRNA was identified by analyzing RNA-seq data from a primary prostate cancer sample in our cohort.

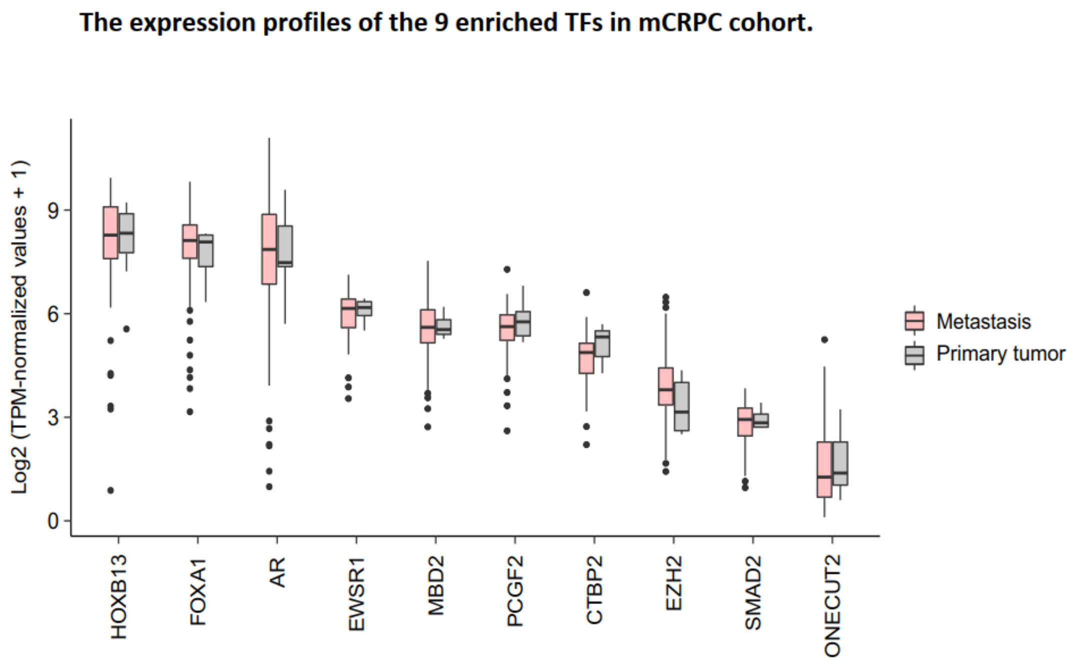


**Supplementary Fig. 4. The expression profiles of the 9 enriched TFs in our mCRPC cohort.**

The read counts were normalized using the TPM method. Box plots are presented on a log2 (TPM + 1) scale.

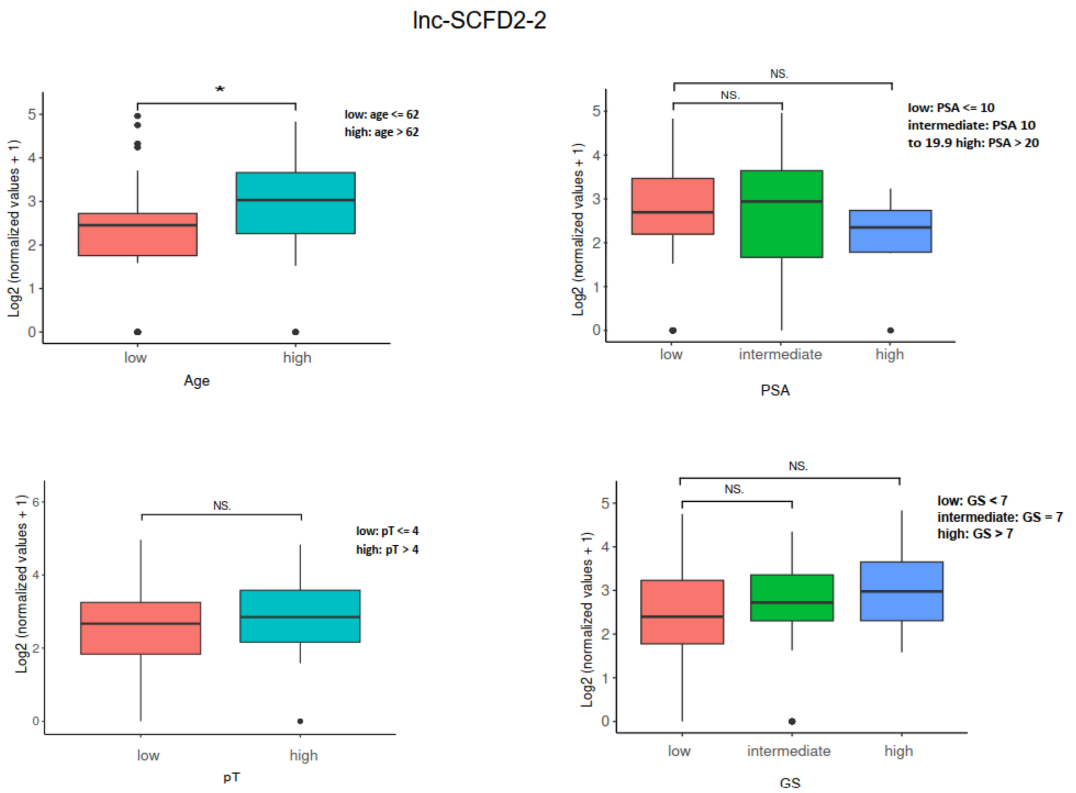
lnc-SCFD2-2
Log2 (normalized values + 1)
low: age <= 62
high: age > 62
Age
low
high
NS.
low: PSA <= 10
intermediate: PSA 10
to 19.9 high: PSA > 20
PSA
intermediate
low: pT <= 4
high: pT > 4
pT
low: GS < 7
intermediate: GS = 7
high: GS > 7
GS

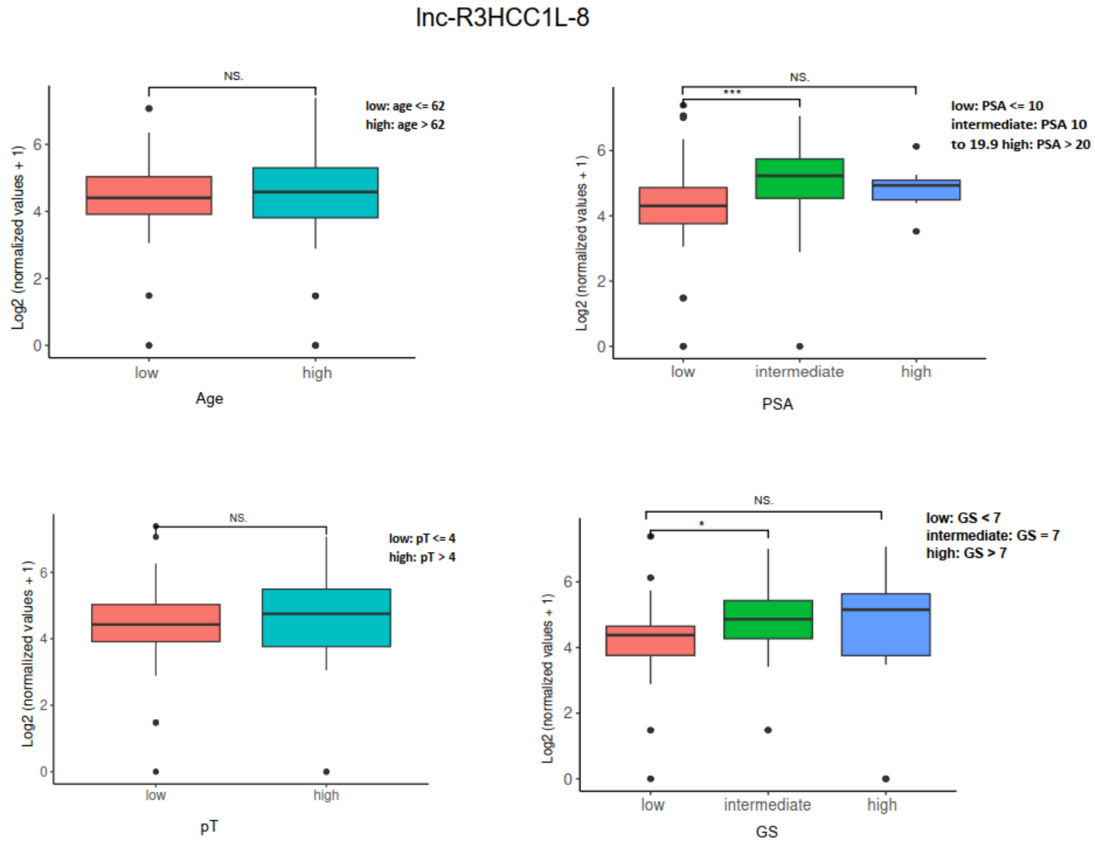
lnc-R3HCC1L-8
Log2 (normalized values + 1)
NS.
low: age <= 62
high: age > 62
Age
low
high
***
low: PSA <= 10
intermediate: PSA 10
to 19.9 high: PSA > 20
PSA
intermediate
low: pT <= 4
high: pT > 4
pT
*
low: GS < 7
intermediate: GS = 7
high: GS > 7
GS

**Supplementary Fig. 5. Associations of the expression of DE-lncRNAs with multiple variables.**

Box plots are showing the association of the expression of two DE-lncRNAs, lnc-SCFD2-2 and lnc-R3HCC1L-8, with age, PSA, pT (pathological T stage), and GS (Glysone score). The raw read counts were normalized using the DESeq2 method. Box plots are presented on a log2 (deseq2 normalized + 1) scale. The cut-off points defining low, intermediate, and high for each variable have been shown on the right side of each plot.
