# Supplementary material for: Identification of long noncoding RNAs with aberrant expression in prostate cancer metastases
Source: Endocr Relat Cancer. 2023 Jun 26;30(8):e220247. doi: 10.1530/ERC-22-0247 (PMC10326635; doi:10.1530/ERC-22-0247)
Supplement: Supplementary Table S8. Univariate Cox regression analysis. [file supplementary_table_8.pdf]

Supplementary Table S8. Univariate Cox regression analysis.

The third quartile was used as cut-off point for high versus low expression.

	lnc-SCFD2-2		lnc-R3HCC1L-8	
**Variable**	**HR (95% CI)**	**P-value**	**HR (95% CI)**	**P-value**
low EXP-level	1.000 (reference)		1.000 (reference)	
high EXP-level	1.758 (1.005–3.078)	0.048	2.284 (1.313–3.974)	0.003
